# Mirk/Dyrk1B mediates G0/G1 to S phase cell cycle progression and cell survival involving MAPK/ERK signaling in human cancer cells

**DOI:** 10.1186/1475-2867-13-2

**Published:** 2013-01-11

**Authors:** Jingchun Gao, Yi Zhao, Yunyi Lv, Yamin Chen, Bing Wei, Jianxin Tian, Zhihai Yang, Fandou Kong, Jian Pang, Jiwei Liu, Hong Shi

**Affiliations:** 1 Department of Obstetrics & Gynecology, First Affiliated Hospital of Dalian Medical University, Zhongshan Road 222, Dalian, Liaoning 116011, China; 2 Department of Oncology, First Affiliated Hospital of Dalian Medical University, Dalian, Liaoning, 116011, China

**Keywords:** Mirk/Dyrk1B, MAPK/ERK, siRNA, Cell cycle, Cancer cells

## Abstract

**Background:**

Mirk/Dyrk1B contributes to G0 arrest by destabilization of cyclin D1 and stabilization of p27kip1 to maintain the viability of quiescent human cancer cells, and it could be negatively regulated by mitogenic-activated protein kinase (MAPK)/extracellular signal-regulated kinase (ERK) signaling. This study was performed to investigate the effect of Mirk/Dyrk1B on cell cycle and survival of human cancer cells involving MAPK/ERK signaling.

**Methods:**

The correlations between Mirk/Dyrk1B expression and active ERK1/2 detected by western blot in both ovarian cancer and non-small cell lung cancer (NSCLC) cells were analyzed by simple regression. Mirk/Dyrk1B unique phosphopeptides with sites associated with Mirk/Dyrk1B protein were isolated and quantitated by liquid chromatography coupled to tandem mass/mass spectrometry (LC-MS/MS) proteomics analysis. The human cancer cells were treated with small interfering RNAs (siRNAs) and/or U0126, an inhibitor of MEK for indicated duration, followed by investigating the alterations of cell cycle and apoptosis as well as related proteins examined by flow cytometry and Western blot, respectively.

**Results:**

Our study demonstrated the widely expressed Mirk/Dyrk1B proteins in the human cancer cells were positively correlated with the levels of activated ERK1/2. Moreover, Mirk/Dyrk1B protein expressions consistent with the tyrosine autophosphorylated levels in the human cancer cells were increased by U0126 or growth factor-depleted culture. Conversely, knockdown of Mirk/Dyrk1B by siRNA led to up-regulated activation of c-Raf-MEK-ERK1/2 pathway and subsequent changes in cell cycle proteins (cyclin D1, p27kip1), accompanied by increased growth rate and cells from G0/G1 into S of cell cycle which could be blocked by U0126 in a dose-dependent manner, indicating Mirk/Dyrk1B may sequester MAPK/ERK pathway, and vice versa. Whereas, combined Mirk siRNA and U0126 induced cell apoptosis in the human cancer cells.

**Conclusions:**

These data together show that Mirk/Dyrk1B mediates cell cycle and survival via interacting with MAPK/ERK signals and simultaneous inhibition of both pathways may be a novel therapeutic target for human cancer.

## Background

The serine/threonine kinase, Mirk/Dyrk1B is expressed in few normal tissues, but in skeletal muscle and many types of human cancers [1]. Mirk/Dyrk1B has the ability to auto-phosphorylate on tyrosine activating itself and then phosphorylate other substrates on serine and threonine; therefore, it has been categorized as a dual function kinase. One role of Mirk/Dyrk1B in skeletal muscle differentiation after a stress signal of serum deprivation is to block cycling myoblasts in the G0 quiescent state [2] by phosphorylation of the cell cycle regulators cyclin D1 and CDK inhibitor p27kip1 [3,4]. Specificially, phosphorylation by Mirk/Dyrk1B at a conserved ubiquitination site Thr288 initiates proteolysis of cyclin D1, while p27kip1 was stabilized following phosphorylation by Mirk/Dyrk1B at Ser10. As normal cells in quiescence activate pathways that protect them from metabolic stress, the subpopulation of tumor cells is likely to utilize similar pathways to survive within the tumor microenvironment. Recently, it has been reported that Mirk/Dyrk1B functions independently and additively to regulate the exit of cancer cells from quiescence through regulating cyclin D turnover and p27kip1 stabilization in colon, pancreatic and ovarian cancer cells shown by Mirk-depletion studies [5-7]. As a result, the quiescent cancer cells depleted of Mirk/Dyrk1B out of G0 entering into the cell cycle may enhance cancer cell kill by chemotherapeutic drugs or radiation, while having less effect on normal tissues in which Mirk/Dyrk1B levels are quite low.

Currently, ovarian cancer and NSCLC are among the leading causes of cancer-related mortality in the world [8]. The presence of drug-resistant, higher proportion of quiescent cancer cells with high clonogenic capacity and tumorigenicity is known to increase recurrence of human cancer and decrease patient survival. Our recent studies have found Mirk/Dyrk1B is overexpressed in a wide spectrum of cell lines and tumor specimens of ovarian and lung cancers [9,10]. Furthermore, knockdown Mirk/Dyrk1B by small interfering RNA (siRNA) induced cell apoptosis and increased sensitivity of human cancer cells to conventional chemotherapeutics *in vitro*[9-12]. More recently, Mirk/Dyrk1B has been found to contribute G0 arrest and maintain viability of the quiescent cancer cells via mediating cyclin D1 and p27kip1 which is associated with reduction of reactive oxygen species (ROS) [5,13], therefore, we hypothesize that Mirk/Dyrk1B pathway may be a novel target for overcoming the drug-resistance and recurrence of various human cancers.

As well as Mirk/Dyrk1B regulating cell cycle-related proteins cyclin D1 and p27kip1, it has been demonstrated that blocking mitogenic-activated protein kinase kinae (MEK) - extracellular signal-regulated kinase (ERK) signaling pathway increases Mirk abundance by up-regulating Mirk/Dyrk1B transcription in either myoblast or colon cancer cells [2], suggesting the possible involvement of mitogenic-activated protein kinase (MAPK)/extracellular signal-regulated kinase (ERK) signaling in Mirk/Dyrk1B functions. However, to date, insufficient data regarding the interaction between Mirk/Dyrk1B and MAPK/ERK in human cancer cells are available, and the mechanisms involved need to be elucidated. In this study, we have identified that the expressed Mirk/Dyrk1B in both ovarian cancer and NSCLC cells is positively correlated with expression of activated ERK1/2. Mirk/Dyrk1B mediates G0/G1 to S of cell cycle and cell survival involving MAPK/ERK pathway in the human cancer cells. It may be a novel target via inhibiting both Mirk/Dryk1B and MAPK/ERK signals for the treatment of human cancer.

## Materials and methods

### Antibodies

The rabbit polyclonal Mirk/Dyrk1B antibody (C-term, AP7538b) was purchased from Abgent (San Diego, CA, USA). Anti-p27kip1, anti-cyclin D1, anti-ERK1/2, and goat anti-mouse IgG horseradish peroxidase (HRP)-conjugated secondary antibody were purchased from Santa Cruz Biotechnology (Santa Cruz, CA, USA). Anti-poly (ADP-ribose) polymerase (PARP) and anti-phosphotyrosine (pY) purchased from Cell Signaling Technology (Danvers, MA, USA). Anti-C-Raf and -phosphorylated C-Raf (P-C-Raf), anti-phosphorylated ERK1/2 Threonine 202/Tyrosine 204 (P-ERK1/2) were purchased from BD Biosciences PharMingen (San Diego, CA, USA). Anti-β-actin and donkey anti-rabbit IgG HRP-conjugated secondary antibody were purchased from Sigma (St. Louis, MO, USA) and Amersham Biosciences (Piscataway, NJ, USA), respectively.

### Cell lines and cell culture

Human ovarian cancer cell lines used were OV2008, OVCAR3, OVCAR5, SKOV3, MDAH2774, OVCAR10, OV1063, and OVCAR8. Of eight cell lines, SKOV3 and OVCAR3 were purchased from American Type Culture Collection (Manassas, VA, USA); others and all NSCLC cell lines used in this study, such as HCC827, PC-9, H1975, H292, H358, H441, A549, and H1299 were gifts from H. Lee Moffitt Cancer Center and Research Institute, USA. All lines were maintained in DMEM supplemented with 10% heat-inactivated (56°C, 30 minutes) fetal bovine serum (FBS; Invitrogen, Grand Island, NY, USA). Monolayer cultures were incubated at 37°C in a 95% humidified atmosphere air containing 5% CO_2_.

### Small interfering RNA treatment

Cells were reverse transfected with small interfering RNAs (siRNAs) using lipofectamine 2000 transfection reagent (Invitrogen) according to the manufacturer’s instructions. The Mirk/Dyrk1B siRNA duplexes as well as the corresponding nonspecific control siRNA duplexes as described [10] were supplied by Dharmacon (Pittsburgh, PA, USA). For combined treatment, cells were pretreated with U0126, an inhibitor of MEK purchased from CalBiochem-NovaBiochem Corporation (La Jolla, CA, USA) at dose escalation for 1 h followed by combination with a constant 20nM dose of siRNAs. Through indicated duration of each treatment, cells treated were harvested and saved for the following experiments.

### Cell proliferation assay

Cells were plated in 96-well plates, and siRNA transfection was performed for 72 hours as described above. Cellular proliferation was measured by [3-(4,5)-dimethylthiazol-2-yl]-2,5-diphenyltetrazolium bromide (MTT) analysis [14]. Briefly, after cells were washed with PBS, they were incubated in MTT solution for 4 hours and then supplemented with 100 μl of dissolving solution (10% SDS in 0.01 M HCl). The absorbance (optical density units) was measured with a microplate spectrophotometer (Bio-Rad Laboratories, Hercules, CA, USA) with Microplate Manager 5.1 software at wavelengths of 590 nm and 660 nm. Each assay was performed in quadruplicate.

### Flow cytometry analysis

After 72-hour treatment with siRNAs, cells were subjected to flow cytometry analyses of apoptosis. Apoptosis was assayed using Pharmingen PE-conjugated monoclonal active caspase-3 antibody apoptosis kit without modification as described previously [10]. We determined the percentage of cells in G_1_, S, and G_2_/M by propidium iodide staining as described previously [15]. A total of 10,000 cells per experimental condition were used for processing and analysis of fluorescence on Becton-Dickinson FACScan (BD, Franklin Lakes, NJ, USA) using CellQuest software.

### Western blot analysis

Cells were washed twice with cold PBS and lysed with buffer A [10 mM Tris–HCl (pH 7.4), 1% Triton X-100, 0.1% SDS, 150 mM NaCl, 1 mM EDTA, 1 mM dithiothreitol, 0.5 mM phenylmethylsulfonyl fluoride, 10 μg/ml leupeptin, 5 μg/ml aprotinin]. After incubation for 30 minutes on ice, the suspensions were centrifuged (15,000 g for 30 minutes). The supernatants were removed and stored at −80°C until analysis using gel electrophoresis. The protein concentration was determined by Bio-Rad protein estimation assay according to the manufacturer’s instructions. For Western blot analysis, ~60-100 μg of whole cell proteins were separated using 10% or 12% SDS-PAGE and transferred to nitrocellulose membranes. After blocking of the membranes with 10 mM Tris–HCl (pH 7.4), 150 mM NaCl, and 0.1% Tween 20 containing 5% nonfat dry milk at room temperature for 60 minutes, the membranes were incubated with indicated antibodies at 4°C overnight and then with the HRP-conjugated secondary anti-rabbit or anti-mouse antibodies at room temperature for 60 minutes. Each protein was detected using the enhanced chemiluminescence (Amersham Biosciences, Piscataway, NJ, USA) system. β-actin was used as an internal control.

Immunoprecipitations were performed with 500 μg of whole cell protein lysates, using Protein A-agarose (Roche, Indianapolis, IN, USA). Briefly, equal amount of protein lysates were incubated with Mirk/Dyrk1B antibody and normal rabbit IgG used as negative control. After incubation for overnight at 4°C, the immune complexes were precipitated with Protein A-agarose. The immunoprecipitates were washed with lysis buffer according to the manufacturer’s instructions, then separated by SDS-PAGE, and transferred to nitrocellulose membranes followed by incubation of pY or Mirk antibodies for western blot analysis as described above.

### Phosphopeptide immunoprecipation and analysis by liquid chromatography coupled to tandem mass/mass spectrometry (LC-MS/MS)

Phosphopeptide immunoprecipitation for eight NSCLC cell lines: HCC827, PC9, H1975, H292, H358, H441, A549, and H1299 was performed using phosphoscan kit (P-Tyr-100, Cell Signaling) according to the manufacturer’s instructions. Using an immunoaffinity peptide profiling technique, Mirk/Dyrk1B unique phosphopeptides with sites associated with Mirk/Dyrk1B protein were isolated and quantitated by LC-MS/MS proteomics analysis as described previously [16]. Results were subjected to sequest-IPI database searching according to criteria specified by molecular and cellular proteomics/cell signaling technology (MCP/CST). Duplicate samples for each cell line each with two technical runs were then filtered according to a 80% peptide identification probability and a 50% protein identification probability.

### Statistical analysis

Each experiment was repeated three times. Data are presented as mean ± SD. Statview 5.0 software was used for statistical analyses. Statistical comparison among the groups was performed using one-way analysis of variance (ANOVA), followed by the Fisher least significant difference test. The correlations between Mirk/Dyrk1B expression and active ERK1/2 were analyzed by simple regression. Differences were considered to be statistically significant when *P* was less than 0.05.

## Results

### Widely expressed Mirk/Dyrk1B in the human cancer cells is positively correlated with activated ERK1/2

**Figure 1:**
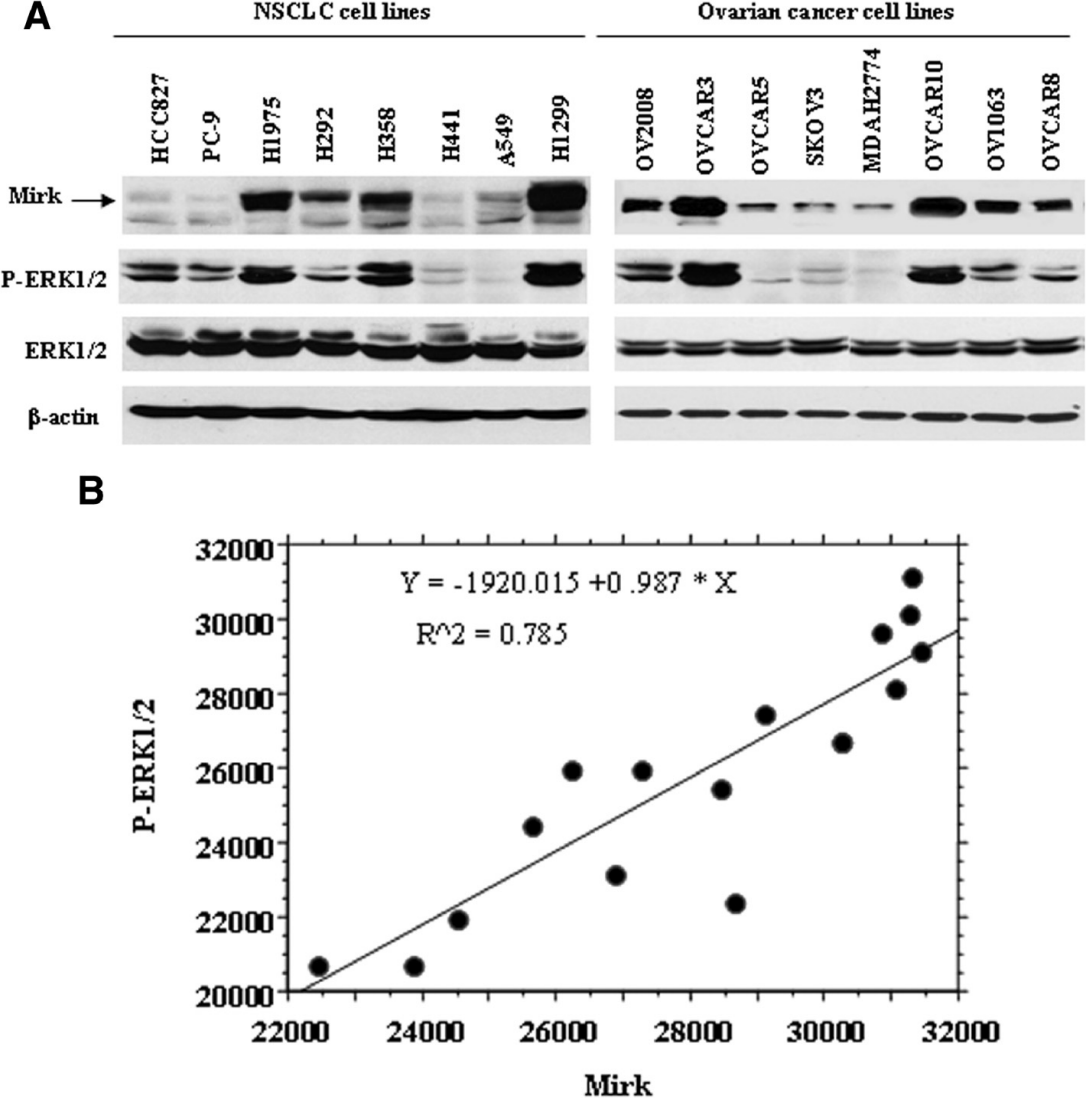
**Widely expressed Mirk/Dyrk1B in the human cancer cells is positively correlated with activated ERK1/2. **(**A**), preotein expressions of Mirk (69 and 71 Kda), ERK1/2 and P-ERK1/2 (42/44 kDa) in ovarian cancer and NSCLC cells were measured by western blot analyses, with equal loading and transfer shown by repeat probing with β-actin (42 kDa). (**B**), the correlation between the protein expressions of Mirk and P-ERK1/2 was analyzed by simple regression. Units, intensity/mm^2^. NSCLC, non-small cell lung cancer; ERK, extracellular signal-regulated kinase.

In this study, we first evaluated protein expression of Mirk/Dyrk1B in both ovarian cancer and NSCLC cell lines. We observed all 16 cell lines were expressed Mirk/DYRK1B protein (Figure 1A). Based on the hypothesis described above that the MAPK/ERK may be involved in Mirk/Dyrk1B function in human cancer, we further examined the expression of both ERK1/2 and P-ERK1/2 in the 16 cell lines (Figure 1A). As shown in Figure 1B, there appears to be positive correlation between the protein expressions of MirkDyrk1B and P-ERK1/2 in all lines (R2 = 0.785 and P < 0.001), suggesting the activated ERK1/2 may be associated with Mirk/Dyrk1B function or kinase activity.

### Enrichment of autophosphorylated Mirk/Drk1B consistent with the protein expression may be mediated by activated ERK1/2

**Figure 2:**
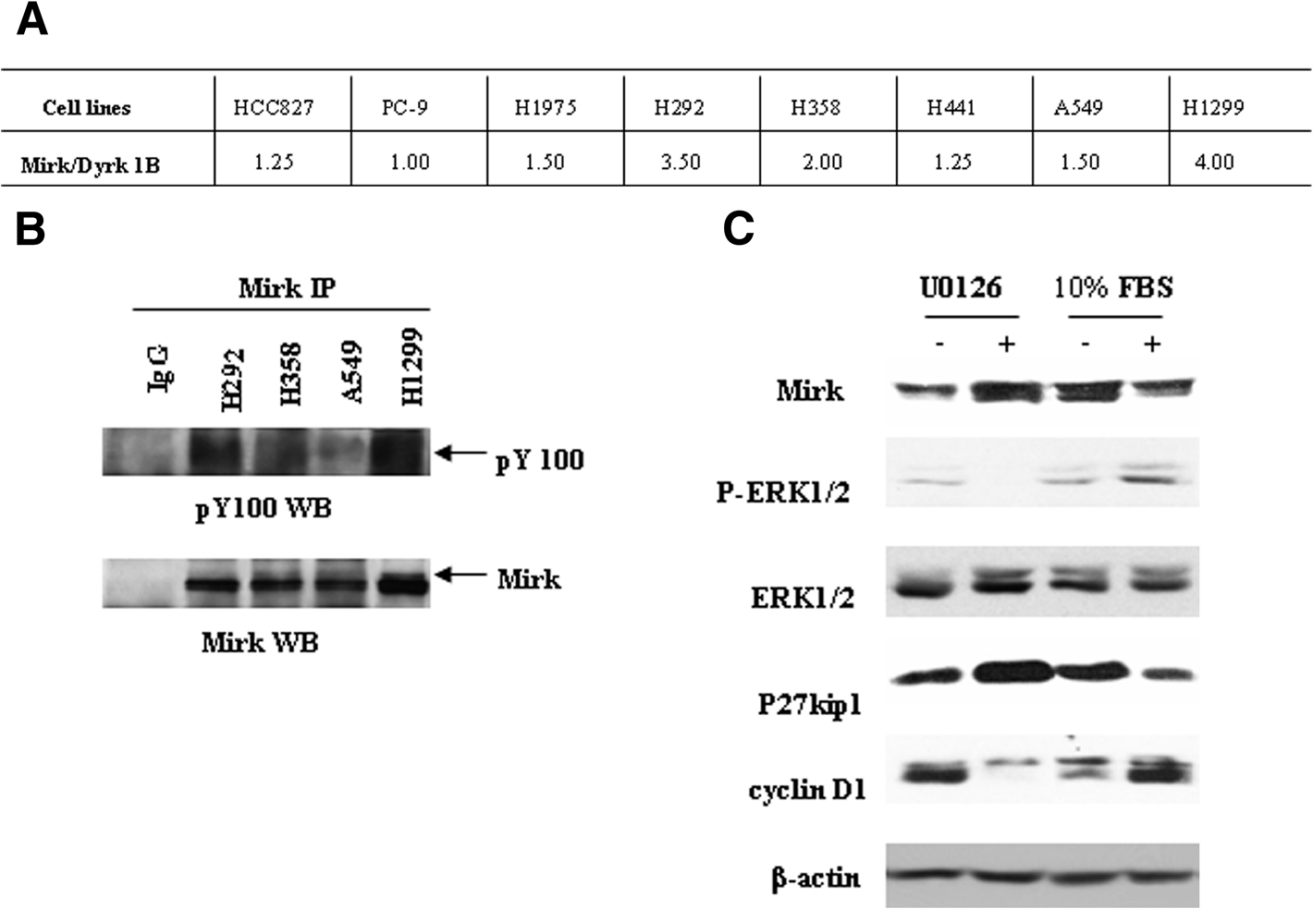
**Enrichment of autophosphorylated Mirk/Dyrk1B consistent with the protein expression may be mediated by activated ERK1/2. **(**A**), the averaged spectral counts corresponding to the phosphotyrosine (pY) autophosphorylation site of Mirk/Dyrk1B in NSCLC cells were analyzed by LC-MS/MS proteomics. (**B**), the pY bands of Mirk/Dyrk1B were detected by Western blot analysis in four lines H292, H358, A549 and H1299 after the cell protein extracts were immunoprecipitated with Mirk antibody and then immunobloted by pY monoclonal antibody. (**C**), H292 cells treated with/without U0126 (10 μM) for 48 h or 10% FBS for 24 h were collected and the protein expressions of Mirk (69 and 71 Kda), ERK1/2 and P-ERK1/2 (42/44 kDa), as well as alterations of cyclin D1 (36 Kda) and p27kip1 (27 kDa) were analyzed by western blot. Equal loading and transfer were shown by repeat probing with β-actin (42 kDa). NSCLC, non-small cell lung cancer; LC-MS/MS, liquid chromatography coupled to tandem mass/mass spectrometry; ERK, extracellular signal-regulated kinase; FBS, fetal bovine serum; IP, immunoprecipitate; WB, western blot.

As part of a phosphoproteomics screen in human cancer cells, we identified peptides corresponding to the pY autophosphorylation site of Mirk/Dyrk1B in NSCLC cells. Figure 2A shown were averaged pY spectral counts across 8 cell lines, of which higher level of pY peptide of Mirk/Dyrk1B were enriched in H1299 cells compared with that in the other cell lines. To further confirm the identified peptides, cell protein extracts out of H292, H358, A549 or H1299 were immunoprecipitated with Mirk/Dyrk1B antibody, and immunobloted by pY and Mirk/Dyrk1B antibodies. The corresponding Mirk/Dyrk1B pY bands were found in all of four lines (Figure 2B). As a control, there was no obvious band in immunoprecipitates prepared with IgG (Figure 2B). There seemed to be positive correlation between the expression of Mirk/Dyrk1B protein and the phosphotyrosine abundance of Mirk/Dyrk1B in NSCLC cells (Figure 1). Therefore, we hypothesize that Mirk/Dyrk1B kinase may be activated via autophosporylation at its phosphotyrosie site. Moreover, consistent with previous report that Mirk/Dyrk1B could be negatively regulated by inhibition of MEK-ERK signaling, in this study western blot analysis also showed that treatment of H292 cells with U0126 for 48 h induced a dose-dependent increase in Mirk/Dyrk1B protein levels (Figure 2C and data not shown), and exposure of H292 cells to 0% FBS for 24 h resulted in up-regulation of Mirk protein levels compared with that to 10% FBS, indicating the increased Mirk possibly mediated by activated ERK1/2 to function cell growth and survival in human cancer.

### Mirk/Dyrk1B regulates progression from G0/G1 to S phase of the cell cycle via MAPK/ERK signaling

**Figure 3:**
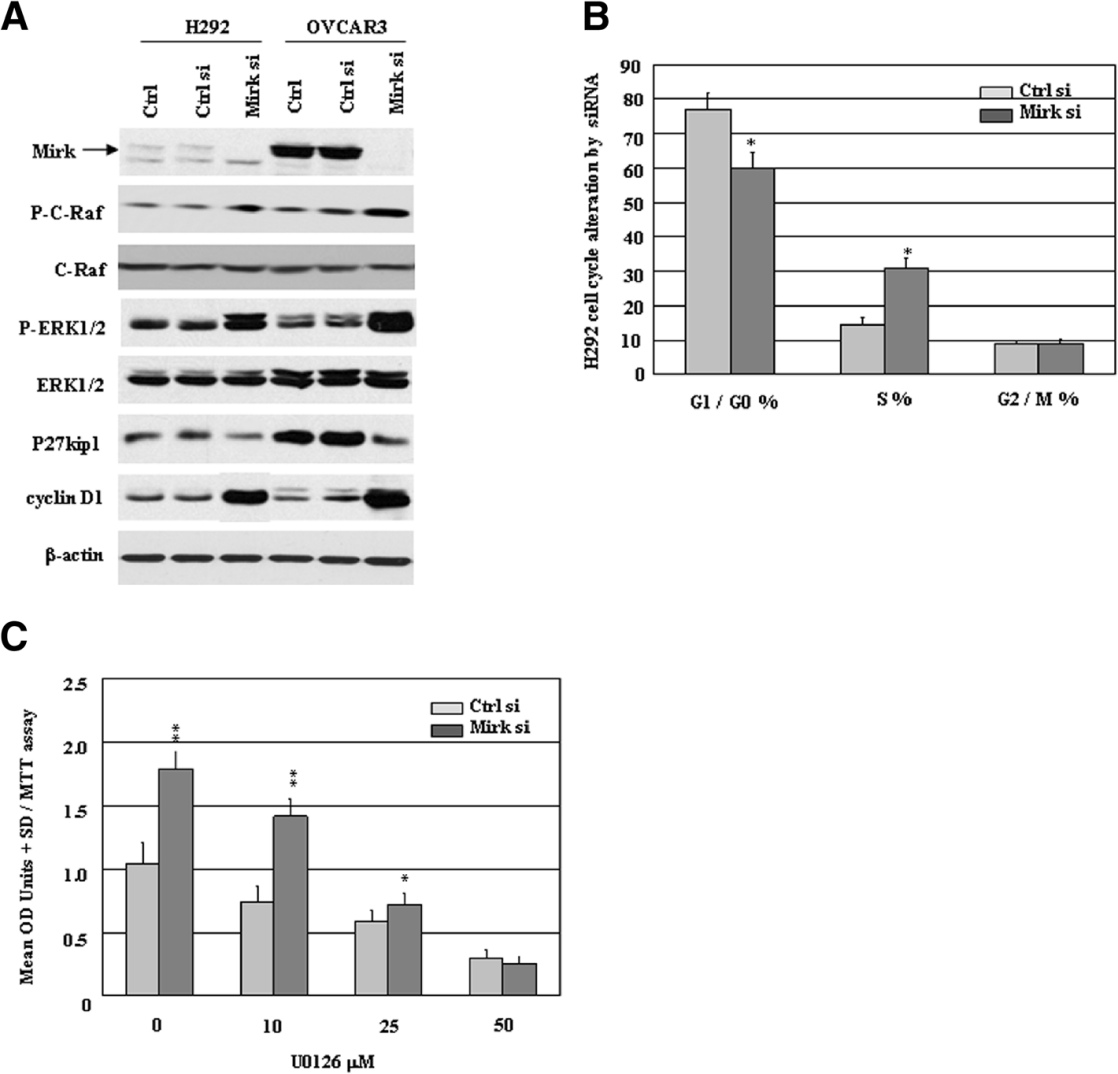
**Mirk regulates G0/G1 to S phase cell cycle via MAPK/ERK signaling. **To investigate this possibility that Mirk expression is associated with activated ERK1/2, the human cancer cells treated with 20 nM siRNAs alone or in combination with a range of concentrations of U0126 for 72 h were collected, and followed by (**A**) the protein expressions of Mirk (69 and 71 Kda), ERK1/2 and P-ERK1/2 (42/44 kDa), C-Raf and P-C-Raf (74 kDa), as well as alterations of cyclin D1 (36 Kda) and p27kip1 (27 kDa) in H292 or OVCAR3 cells were measured by western blot analyses. Equal loading and transfer were shown by repeat probing with β-actin (42 kDa); (**B**) H292 cells stained with propidium iodide were subjected to flow cytometry analysis; and (**C**) H292 growth rate was measured by MTT assay. *, P < 0.05; **, P < 0.01 compared with control. siRNA, small interfering RNA; MAPK, mitogenic-activated protein kinase; ERK, extracellular signal-regulated kinase. Ctrl, no siRNA; Ctrl si, control siRNA; Mirk si, Mirk siRNA.

As Mirk in skeletal muscle not only blocks cycling myoblasts in G0 quiescent state for differentiation but also limits apoptosis in fusing myoblasts, it may regulate cell cycle and survival through the similar mechanisms in human cancer. To investigate the effects and mechanisms of Mirk involving MAPK/ERK pathway in human cancer cells, the upstream or downstream signals of ERK1/2 were first determined in a representative panel of H292 and OVCAR3 cells treated with 20 nM siRNA duplexes, with Mirk siRNA #4 targeting Mirk for 72 h as reported previously [10]. As shown in Figure 3A, exposure of both cell lines to Mirk siRNA was associated with knockdown of Mirk, up-regulation of P-C-Raf, P-ERK1/2 as well as cyclin D1, and reduction of p27kip1, compared with that shown with control siRNA. We next investigated the effects of altered MAPK/ERK pathway by Mirk knockdown on the human cancer cells, the H292 or OVCAR3 cells treated with 20 nM siRNA for 72 h were collected, stained with propidium iodide, and subjected to flow cytometry analysis. Knockdown Mirk by siRNA resulted in S phase accumulation of cells accompanied by decreased cell number of G1/G0 phase in H292 or OVCAR3 (Figure 3B and data not shown). Consequently, Mirk siRNA treatment increased growth rate of the human cancer cells which could be blocked by U0126 (Figure 3C and data not shown), suggesting knockdown Mirk-induced cell cycle alteration may be involving MAPK/ERK pathway.

### Mirk modulates cell survival associated with activation of MAPK/ERK

**Figure 4:**
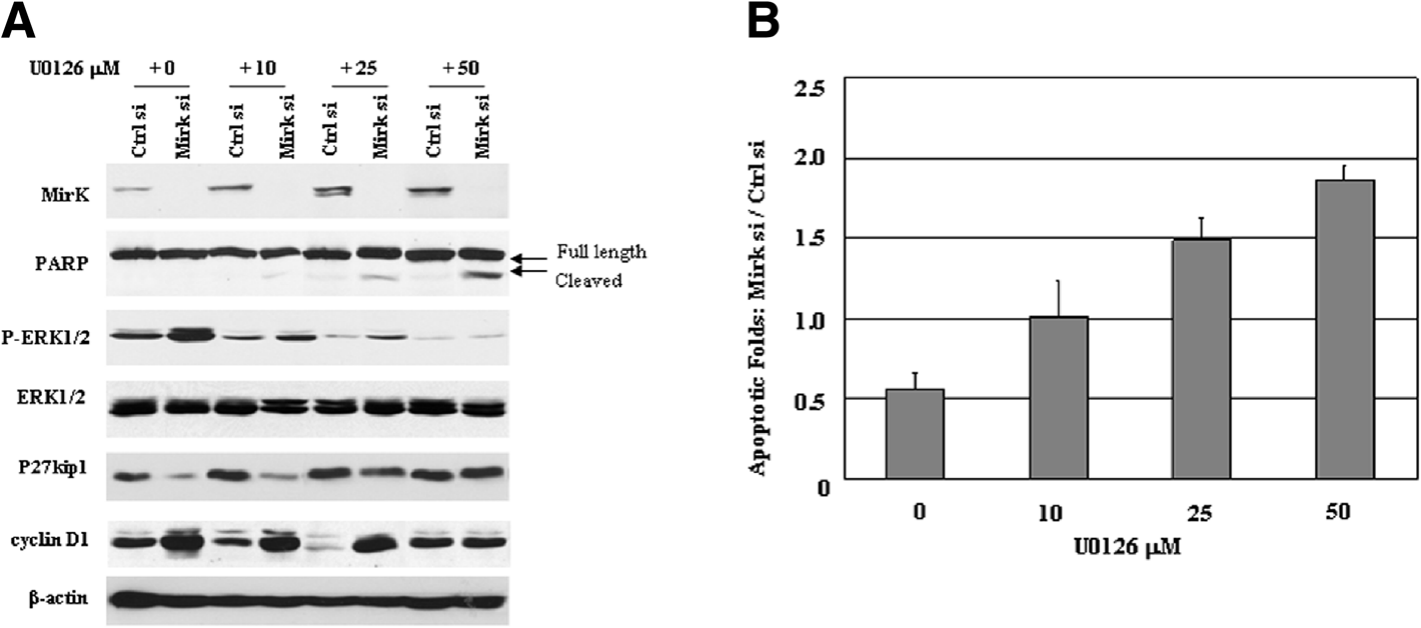
**Mirk modulates cell survival associated with activation of MAPK/ERK. **H292 cells exposed to 20 nM siRNAs with/without a range of concentrations of U0126 for 72 h were collected, and followed by (**A**) the protein expressions of Mirk (69 and 71 Kda), PARP (full length 116 kDa and cleaved 89 kDa), ERK1/2 and P-ERK1/2 (42/44 kDa), as well as alterations of cyclin D1 (36 Kda) and p27kip1 (27 kDa) were measured by western blot analyses. Equal loading and transfer were shown by repeat probing with β-actin (42 kDa); (**B**) cell apoptosis (fold of control) evidenced by positive cells with active caspase-3 was assessed by flow cytometry analysis. MAPK, mitogenic-activated protein kinase; ERK, extracellular signal-regulated kinase; PARP, poly(ADP-ribose) polymerase. Ctrl si, control siRNA; Mirk si, Mirk siRNA.

To further determine the effects of MAPK/ERK pathway involved in Mirk modulating cancer cell survival, the H292 cells were treated with 20 nM siRNAs with/without U0126 in gradient for 72 h followed by western blot and flow cytometry analyses, respevtively. As shown in Figure 4A, U0126 blocked knockdown Mirk-induced ERK1/2 activation, as well as alteration of downstream signals p27kip1 and cyclin D1 in H292 cells. Interestingly, Mirk siRNA combined with U0126 led to increased cell apoptosis evidenced by PARP cleavage (Figure 4A) and positive cells with active caspase-3 (Figure 4B). Taken together, these results suggest that MAPK/ERK may be a novel pathway with which Mirk interacts to serves as an antiapoptotic factor in the human cancer cells.

## Discussion

Mirk/Dyrk1B is a member of a conserved family of serine/threonine kinases that are actived by intramolecular tyrosine phosphorylation which mediate maturation in defferent tissues: Mirk in skeletal muscle, Dyrk1A in brain, etc. [1]. Previous studies show that Dyrk1A activity was increased by binding to 14-3-3 protein [17], and is mediated by autophosphorylation at a C-terminal serine [18], which is not conserved in Mirk. The possible YxY activation domain of Mirk within the conserved kinase region has been known to be intramolecularly phosphorylated only during translation, and the mature members of Mirk family have only serine/threonine kinase activity [19]. Whereas, in this study we utilized a phosphoproteomics screen to identify peptides corresponding to the tyrosine autophosphorylation site of Mirk/Dyrk1B in the human cancer cells and demonstrated a positive correlation between the expression of Mirk protein and the phosphotyrosine abundance of Mirk/Dyrk1B. This suggests that activation of Mirk/Dyrk1B kinase in the deregulated cancer cells may be mediated by tyrosine autophosphorylation, although further study is required.

Mirk/Dyrk1B is also an arginine-directed serine/threonine kinase which has limited expression in normal tissue with highest expression seen in skeletal muscle, heart, tests, and brain. Initially, most of the studies of Mirk/Dyrk1B have been conducted using myogenesis as a model system. It has been known that Mirk/Dyrk1B functions as a transcription factor activator in muscle differentiation. In cultured myoblasts, mitogen deprivation increased Mirk/Dyrk1B protein levels predominantly via transcriptional mechanisms regulated by RhoA and Cdc42, and to a lesser extent by Rac1 [2]. Inhibition of MAPK/ERK activity by removal of serum mitogen [20] or by addition of the MEK inhibitor increased endogenous Mirk/Dyrk1B protein levels and its promoter construct [2], suggesting Mirk/Dyrk1B induction in muscle differentiation requires not only active Rho proteins but also inhibition of the MAPK/ERK signaling pathway. Consistently, the colon cancer cells has been found to utilize similar pathway as myoblasts through which MEK inhibition activates Mirk/Dyrk1B promoter constructs and increases Mirk/Dyrk1B transcription [2]. In this study, we also found Mirk/Dyrk1B could be up-regulated by serum depleted culture or U0126 treatment in both ovarian cancer and NSCLC cells. Therefore, Our study along with others [2,21] suggests that MAPK/ERK signaling may inhibit Mirk/Dyrk1B transcription and functions in human cancer cells. In addition, our results in the study demonstrate that the widely expressed Mirk/Dyrk1B in both ovarian cancer and NSCLC cells largely correlates the activated ERK1/2. Moreover, we have also found Mirk/Dyrk1B protein levels were increased by inhibition of MEK-ERK1/2, and knockdown of Mirk/Dyrk1B resulted in up-regulation of activated ERK1/2 as well as up/downstream signals. All together indicate the possible interaction between Mirk/Dyrk1B and MAPK/ERK signals in human cancer cells. Namely, Mirk/Dyrk1B may sequester MAPK/ERK, and vice versa in either myogenesis or cancer.

In the past decade, growing evidence has demonstrated that knockdown Mirk/Dyrk1B could induce cell apoptosis and increase sensitivity of various human cancer cells to conventional chemotherapeutics. Furthermore, the study in osteosarcoma demonstrates that the overall survival rate of patients is negatively correlated with the levels of Mirk/Dyrk1B protein expression [12]. Our previous studies have also shown Mirk/Dyrk1B function in an NSCLC orthotopic mouse model [10], and the involvement of FoxO1/3A in the Mirk-mediated ovarian cancer cell survival [9]. Recently, studies have focused on the effect of Mirk/Dyrk1B on various human cancer cells arrested in a reversible quiescent state (G0-G1) to undergo DNA repair or survive suboptimal growth conditions [6,7,13]. Moreover, Mirk/Dyrk1B, through regulating cyclin D turnover and p27 stabilization, functions independently and additively to regulate the exit of cancer cells from quiescence G0 or early G1 into S and G2/M of cell cycle [6]. Although it has been reported that Mirk could be negatively regulated by MAPK/ERK in colon cancer, our study is the first to show the possible interaction between Mirk/Dyrk1B and MAPK/ERK signals or sequestering with each other in both ovarian cancer and NSCLC cells. In this study, knockdown of Mirk/Dyrk1B by siRNA in either ovarian cancer cells or NSCLC cells led to up-regulated activation of c-Raf-MEK-ERK1/2 pathway, accompanied by increased growth rate and cells from G0/G1 into S of cell cycle which could be blocked by U0126 in a dose-dependent manner. Furthermore, combined Mirk siRNA and U0126 induced cell apoptosis in the human cancer cells. All of above suggest that Mirk/Dyrk1B may mediate cell cycle and cell survival through interacting with MAPK/ERK pathway in human cancer.

Taken together, the widely expressed Mirk/Dyrk1B in the human cancer cells is positively correlated with the levels of activated ERK1/2. Mirk/Dyrk1B mediates G0/G1 to S of cell cycle and cell survival in both ovarian cancer and NSCLC cells may be associated with MAPK/ERK signaling. Therefore, simultaneous inhibition of Mirk/Dyrk1B and MAPK/ERK may be a novel target for treatment of human cancer.

## Abbreviation

ERK: In Mirk-mediated cancer cell cycle and survival.

## Competing interests

The authors declare no conflict of interest.

## Authors’ contributions

JG, HS, YZ, and JL designed research; JG, YZ, YL, YC, BW, JT, and ZY performed research; JG, YZ, FK, and JP analyzed data; JG, YZ, JL, and HS wrote the paper. All authors read and approved the final manuscript.
